# Efficacy and safety of secondary procedures for maintaining arteriovenous hemodialysis access patency: protocol for a systematic review and network meta-analysis

**DOI:** 10.1186/s13643-020-01435-1

**Published:** 2020-08-21

**Authors:** Mark Rockley, Sudhir Nagpal, Ashish Gupta, Derek J. Roberts

**Affiliations:** 1 University of Ottawa, Ottawa, ON K1N6N5 Canada; 2 Division of Vascular and Endovascular Surgery, Department of Surgery, The Ottawa Hospital - Civic Campus, Ottawa, ON K1Y4E9 Canada; 3 Division of Angiography and Interventional Radiology, Department of Radiology, The Ottawa Hospital - Civic Campus, Ottawa, ON K1Y4E9 Canada

**Keywords:** Angioplasty, Stent, Hemodialysis access, Arteriovenous, Patency

## Abstract

**Background:**

Arteriovenous (AV) hemodialysis access creation is recommended by international guidelines as the preferred method of hemodialysis access. However, most AV access sites will require revision to maintain patency. Although several treatment options exist, many have not been directly compared. We intend to compare the relative effectiveness of methods to maintain post-intervention primary patency of failing AV access.

**Methods:**

We will search EMBASE, MEDLINE, CENTRAL, trial registries, the grey literature, and ancestry and citation search from January 1977 to present, for randomized controlled trials comparing interventions to maintain primary patency of AV access. Two investigators will independently and blindly review all identified citations and extract data from included studies. The primary outcome is the primary patency 6 months after intervention. Secondary outcomes include immediate technical and functional success, reinterventions, patency, and mortality. Risk of bias, subgroup analyses, and sensitivity analyses are planned.

**Discussion:**

There are a number of treatment modalities for the management of failing AV access. However, most modalities have only been directly compared with plain old balloon angioplasty, and currently synthesized evidence focuses on individual pairwise comparisons. In light of the lack of comprehensively synthesized evidence and clinical equipoise, our study intends to synthesize currently available evidence though it is unclear which treatment modality is most effective.

**Systematic review registration:**

PROSPERO ID CRD42020148224

## Background

### Rationale

The prevalence of severe chronic kidney disease in North America as defined by the Kidney Disease: Improving Global Outcomes (KDIGO) has been reported to be as high as 6%, and many of these patients subsequently require hemodialysis [1]. Since the Fistula First Initiative began incentivizing arteriovenous (AV) access for hemodialysis, the majority of eligible patients starting dialysis will receive an arteriovenous fistula (AVF) [2]. Unfortunately, the failure rate of AVFs (due to lack of maturation, stenosis, or occlusion) remains high, estimated at 60% at 1 year in a recent meta-analysis of cohort studies [3]. Hemodialysis AV access is not only common but also expensive. Medicare pays $2.8 billion annually for arteriovenous access-related costs in the elderly, 83% of which experience primary failure within the first year of access creation [4]

As a result, several open surgical and endovascular procedures have been developed to maintain failing AV accesses. The most common is plain old balloon angioplasty (POBA), but post-intervention failure remains common [5]. This high failure rate prompted new technological developments intended to improve treatment effectiveness such as specialized cutting balloons and drug-eluting balloons [6]. While stents are used extensively in arterial atherosclerosis, their use in the treatment of stenoses in AVFs is less common [7]. Similar to angioplasty, there are also several subtypes of stents beyond standard bare-metal stents, including drug-eluting and fabric-covered [8]. Although open surgical revision of existing access sites may also be performed, this is becoming less common given the various minimally invasive options [9].

POBA is repeatedly used as a common comparator in many randomized controlled trials (RCTs) investigating newer technology [10]. Previous pairwise meta-analyses have evaluated evidence comparing POBA with cutting balloons [11, 12], drug-eluting balloons [13, 14], bare metal stenting [15], and stent grafts [16]. While these analyses often demonstrate that POBA is inferior to these other comparator interventions, there are no meta-analyses that compare the relative effectiveness between the newer technology comparator interventions [17]. Because there is minimal RCT evidence directly comparing these newer technologies, simple pairwise meta-analysis cannot provide additional insight into comparisons of these new approaches. However, a network meta-analysis could potentially guide clinicians in choosing between the many treatment options until further RCTs directly comparing these various approaches become available.

Currently synthesized evidence is insufficient to direct practical clinical decision-making, and the current variation in operator preference of treatment modality suggests ongoing clinical equipoise. We propose to conduct a systematic review and network meta-analysis to evaluate the effectiveness of the various interventional methods to maintain AV access patency following treatment.

### Objectives

The primary objective of this systematic review and network meta-analysis will be to determine the relative effectiveness of methods to maintain primary patency of failing arteriovenous access 6 months post-intervention. Secondary objectives will compare the immediate functional and technical success, in addition to primary assisted patency, secondary patency, lesion primary patency, and mortality as defined in the “Outcomes and prioritization” section.

## Methods

### Eligibility criteria

#### Study designs

The eligibility criteria are summarized in Table 1. We will include both randomized controlled trials (RCT) and quasi-randomized controlled trials and perform a sensitivity analysis to determine if conclusions are robust to study design variances. Non-randomized controlled trials, interrupted time series studies, case-control studies, controlled before-after studies (CBA), prospective or retrospective cohort studies, cross-sectional studies, and case reports will be excluded. Data from abstracts of eligible studies that have not been published in full manuscript form will be included, and we will perform sensitivity analyses to assess whether inclusion of unpublished trials affects the results.

**Table 1:** Summary of study selection criteria

**Study design**	
Randomized controlled trials	
Quasi-randomized trials	
**Participants**	
Age 18 years or older	
Stenosis of arteriovenous fistula or graft for hemodialysis	
Procedure on AV hemodialysis circuit stenosis	
**Interventions**	
Performed on AV circuit (anastomosis to peripheral-central vein confluence)	
Open or endovascular procedures, including the following: Open surgical revision Plain balloon angioplasty Cutting balloon angioplasty Drug-eluting balloon angioplasty Drug-eluting stenting Bare-metal stenting Venous atherectomy Covered stent grafting	
**Outcomes**	
Primary (essential): Primary AV circuit patency at 6 months	
Secondary (optional): Initial technical result Functional success within 6 months Lesion-specific primary patency at 6 months Primary assisted AV circuit patency at 6 months Cumulative (secondary) AV circuit patency at 6 months Mortality at 6 and 12 months	
**Timing**	
Outcome determination: immediate to 12 months	

#### Participants

We will include studies examining adults (age 18 or older) who received a procedure for a stenotic lesion in an AV hemodialysis circuit. Studies evaluating both AV fistulas and peripheral AV grafts will be included, and differences in outcomes between these patient cohorts will be evaluated in subgroup analyses. Investigations on specialized peripheral AV access with central drainage, such as the Hemodialysis Reliable Outflow (HeRO®) Graft, will be included and assessed for a differential effect using sensitivity analysis. Studies evaluating interventions on occluded AV accesses, including clot-debulking procedures such as thrombolysis or thrombectomy, will be excluded.

#### Intervention and comparators

We will examine studies investigating interventions on AV circuits extending from AV anastomotic lesions to the proximal aspect of the peripheral venous drainage into central veins (i.e., subclavian, jugular, or brachiocephalic). Studies that included subjects with interventions on only central veins or accessory venous embolization will be excluded. If studies include interventions on both peripheral and central veins, the data for outcomes on peripheral interventions only will be included. The venous intervention must be the primary purpose of the intervention, excluding concurrent arterial procedures. Open surgical and endovascular interventions will be included. Potentially eligible treatment methods include open surgical revision, POBA, cutting balloon angioplasty, drug-eluting balloon angioplasty, drug-eluting stenting, bare metal stenting, venous atherectomy, and covered stent grafting. Open surgical procedures must be performed to maintain the existing access; any procedures involving the addition of new graft or additional vein will be considered creation of a secondary procedure and will be excluded.

#### Outcomes

The intent of treatment to maintain AV access is to continue using the specific AV access site. However, the functional use of an AV access site can be difficult to define and ascertain. Therefore, the anatomic patency is often reported as the surrogate outcome. Patency is defined as sustained flow through the AV access site, often reported with qualifiers to distinguish whether additional procedures were required to maintain or restore ongoing blood flow through the access. Because the failure of treatments on AV access can be exceedingly high, the reported outcome follow-up duration is often relatively short term at 6 months post-intervention patency [18].

The primary outcome of our proposed study is circuit primary patency after 6 months post-intervention. Immediate technical and functional success will be secondary binary outcomes. Primary assisted patency, access cumulative (secondary) patency, and lesion-specific primary patency following intervention are secondary outcomes at 6 months. All outcomes are defined in detail in the “Outcomes and prioritization” section.

#### Timing

Patency outcomes will occur at 6 months post-intervention. Immediate technical and success will be recorded immediately following intervention, and functional success will be defined as any functional usability of the access as the sole method of hemodialysis following the secondary procedure within 6 months, given that no interval secondary procedures were performed.

#### Setting

There are no restrictions regarding setting of the study.

#### Language

This study has no language restrictions.

### Information sources

A literature search strategy using medical subject headings and text words has been developed by an investigator with graduate-level training in epidemiology (MR) and another with doctorate-level training in evidence synthesis and knowledge translation (DJR) in consultation with an information scientist (HLR). We will search MEDLINE (OVID interface), EMBASE (OVID interface), Web of Science, Scopus, and the Cochrane Central Register of Controlled Trials (Wiley interface). We will also search the bibliographies of all included trials and any relevant review articles identified during the course of the search. OpenGrey will be interrogated for unpublished relevant literature.

### Search strategy

Both qualitative and quantitative studies will be assessed. All searches will be limited by date of publication (January 1977–present). The starting year of 1977 has been chosen as the first in-human angioplasty was performed that year. No language limit will be placed on the search. The search strategy and syntax will be generated by an information scientist and knowledge translation scientist (DJR), both of whom have extensive systematic review experience. Please see Appendix 1 for the proposed MEDLINE search strategy and Appendix 2 for the proposed EMBASE search strategy. The PROSPERO database has been searched, and no similar ongoing or recently completed systematic reviews on this topic have been performed.

### Study records

#### Data management

Literature search results will be aggregated in EndNote, including where duplicate articles will be removed. The results will then be uploaded to the Distiller SR software.

The two screening authors will independently and blindly screen titles and abstracts resulting from the combined search of all selected databases. The full text of an article will be obtained for any articles that one of the investigators felt appeared to potentially meet eligibility criteria. For these articles, the full text will subsequently be screened for eligibility. Any reasons for exclusion following full text screening will be explicitly documented and listed in an appendix.

Once both reviewers have created a complete list of eligible articles, the lists will be compared. Discrepancies in article selection will be addressed with discussion with a third-party author experienced in systematic review conduct. The authors will not be blinded to the journals of publication.

#### Data collection process

A standardized form created in Microsoft Excel will be used as the data collection method. Both reviewers will have a separate form for each article, which will be compared for consistency after data collection has been completed. Any discrepancy will be resolved through discussion and re-review of the article or consultation with a third independent author. Study authors will be contacted using contact information available with the publication to resolve unclear or inadequate reporting of data, and 1 month will be allowed to provide the additional necessary details.

If only longer-term results are presented in text and our primary outcome of 6-month results are not described in text, we will seek to determine the 6-month results based on reported Kaplan-Meier curves and at-risk study population data. If the independent reviewers can confidently ascertain the 6-month results based on the presented data, these results will be used in our analyses.

### Data items

Generic article data will include year of publication, trial design, the number of patients enrolled into both arms, duration of follow-up, financial support sources and involvement, and publication status. Patient-specific data will include estimates of age, sex, indication for intervention, type of AV access, anatomic location of the AV access, and the anatomic location of lesions. Intervention-specific data will include the treatment methods being compared, including type of drug in drug-eluting technology, and balloon pressure in angioplasty procedures, and the use of adjunctive procedures following the initial therapy such as radiotherapy.

Outcome-specific data will include the blinding status of outcome adjudication, the definition of outcomes, immediate technical and functional success, and any patency outcomes.

### Outcomes and prioritization

#### Primary outcome

Outcomes are summarized in Table 1. To be included in the primary analysis, studies must report the circuit primary patency 6 months following reintervention. Primary patency is defined by the Kidney Health initiative as freedom from thrombosis, any subsequent intervention to facilitate, maintain, or re-establish patency, or other study-specific censoring events [19]. The AV circuit includes any of the inflow artery, AV anastomosis, graft if present, and outflow veins.

#### Secondary outcomes

Studies may participate in secondary analyses if they report the following secondary outcomes described below or as defined by the authors.

- Initial technical success
  - Lack of residual stenosis immediately following treatment, as defined by individual studies if between 20 and 50%.
- Functional success
  - Use of the access for hemodialysis as the sole access within 6 months following the secondary procedure, given that no interval secondary procedures were performed.
- Primary assisted patency
  - Freedom from temporary or permanent access circuit occlusion at 6 months, allowing procedures to maintain patency.
- Access cumulative (secondary) patency
  - Freedom from permanent access circuit occlusion at 6 months, allowing procedures to re-establish patency after temporary occlusion.
- Lesion primary patency
  - Freedom from temporary or permanent lesion-specific occlusion or repeat secondary procedure to maintain patency at 6 months.
- Mortality
  - Documented mortality at 6 and 12 months

### Risk of bias of individual studies

We will use the Cochrane Collaboration Risk of Bias 2 tool to assess individual studies for potential risk of bias [20]. The quality domains included in this tool include random sequence generation, allocation concealment, blinding, incomplete outcome data, and selective outcome reporting. Each study will be determined to be at either low or high risk for each category. Alternately, if the report includes insufficient information to determine the level of risk, the category will be labeled as unclear. The risk of bias within each quality domain will be independently and blindly determined by the two reviewers who also performed data extraction, and compared following complete assessment of all studies. Discrepancies will be addressed with discussion with a third-party author. The resulting risk of bias for each study, in each category, will be graphically represented.

### Data synthesis

We will narratively summarize each study in tables and in text. Specifically, we will describe the descriptive statistics of each study’s population, AV access features, study design, treatment methods, measured outcomes, methods of measurement, and intention-to-treat position. Reported values of all collected study outcomes will be summarized in an appendix table.

#### Quantitative synthesis

**Fig. 1:**
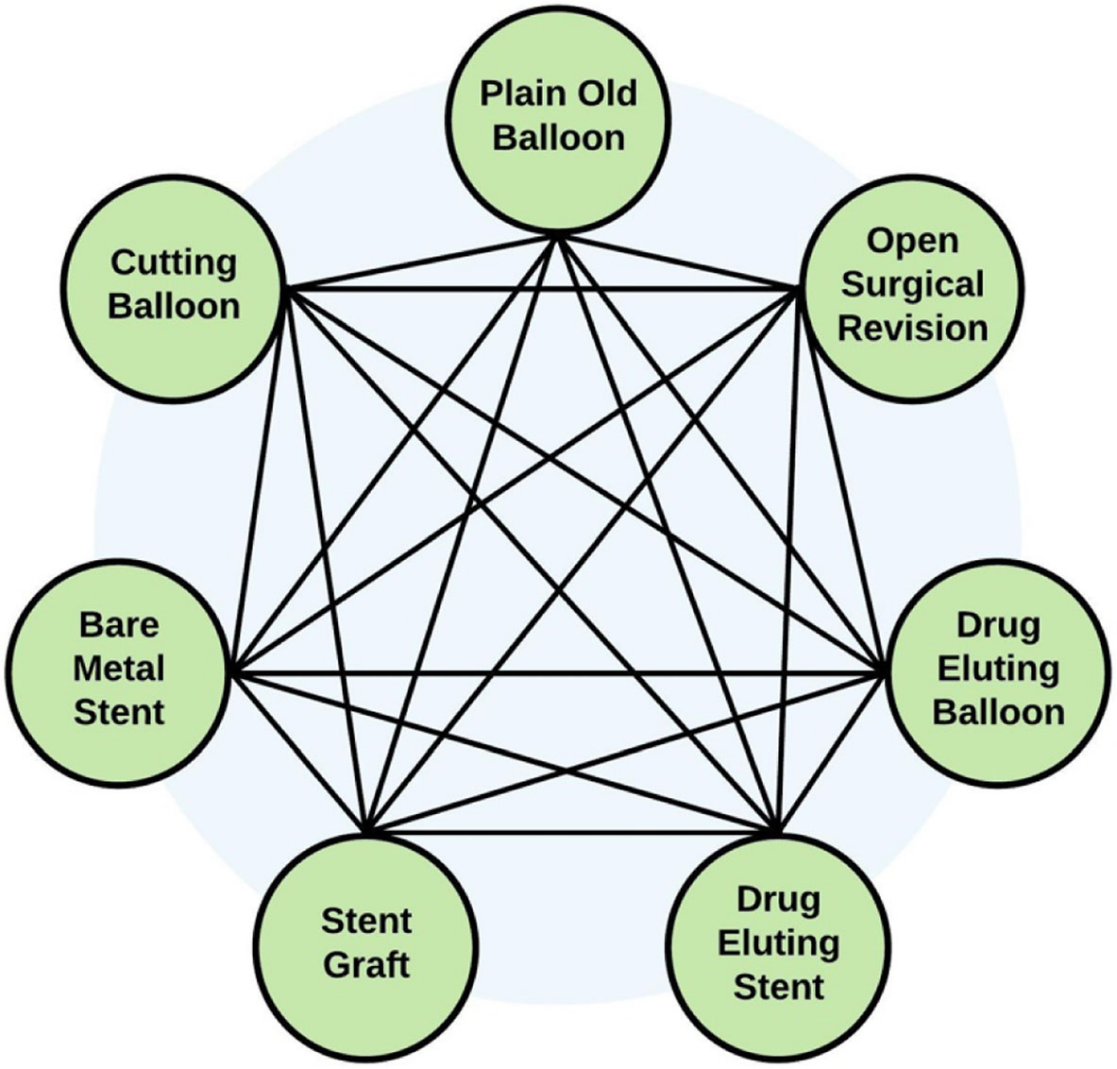
Schematic of potential comparisons between interventions within the network meta-analysis framework

We will assess the studies for meta-analysis appropriateness by evaluating study design, subject eligibility, reported outcomes, methods of outcome assessment, and type of interventions. We will assess for potential network meta-analysis by first generating a node diagram to summarize the direct pairwise comparisons, to identify complete networks of studies. All potential comparisons are presented in Fig. 1, depending on amenability of each comparison to meta-analysis. There is a potential that individual interventions may be omitted from the network comparison if they are not represented by the included studies. If multiple independent networks of studies are identified, separate network meta-analyses will be performed.

If we identify appropriate networks of studies for cumulative analysis, we will perform a Bayesian network meta-analysis. All network meta-analyses will be performed on the NetMetaXL platform utilizing the WinBugs software [21]. Analyses will be performed using random effects models with vague prior parameter distributions, to accommodate subtle heterogeneity. The analyses will be performed using reported ITT results when available.

Convergence of the models will be confirmed using the Brooks-Gelman-Rubin method [22] by repeated Markov chain Monte Carlo analyses, to ensure the error is less than 5% of the standard deviation of the parameter estimates and variance between studies. Estimated measurement effects will be reported as odds ratios with 95% confidence intervals. The unit of analysis will be access sites; one subject may theoretically lend multiple units of analyses if they have multiple AV access sites requiring intervention. Clustering of data for purposes of meta-regression will not be considered due to anticipated low number of studies and variable reporting patterns, resulting in statistical limitations. For the primary outcome, estimated pairwise comparisons within the networks will be described in a league table and graphically represented in a forest plot, and probability bars estimating the likelihood of treatment rank will be presented for each rank in the respective network. We will report the a priori subgroup and sensitivity analyses of the primary outcome. We will report estimated measurement effects of all secondary outcomes using a league table and forest plot where acceptable. Potential subgroup analyses will also include type of AV access, recurrent disease, and location of disease, as described below. Sensitivity analyses will include study risk of bias, study design, unpublished studies, and maturation state of AV access.

#### Issues relating to data quality

In cases of unclear or inadequate data reporting, authors will be contacted for further data using contact information provided by the publishing journal. For trials that did not report on an intention-to-treat basis, or are otherwise at unique risk for bias, sensitivity analysis will be used to assess the effect of inclusion of these trials. Trials will not be excluded due to number of participants; however, the relative effect of the size of trials will be assessed using funnel plots.

We will evaluate inconsistency within the network meta-analysis by evaluating the deviance residuals in fitted consistency and inconsistency models as suggested by the National Institute for Health and Care Excellence [23]. The posterior mean deviance of estimates in the consistency and inconsistency models will be described in an inconsistency plot.

#### A priori subgroup analyses

If multiple studies with homogenous outcomes are reported within the following subgroups, planned subgroup analyses of the primary outcome include the following:

- Type of AV access
  - Fistula
  - Graft
- Recurrent disease
- De novo lesions
- Recurrent lesions
- Anatomic location of arterial intervention
- Anastomotic
- In-graft (for situations involving AV grafts)
- Distal peripheral vein (including cephalic and basilic veins)
- Proximal peripheral vein (including cephalic arch)

#### A priori sensitivity analyses

Planned sensitivity analyses include the following:

- Studies at high risk of bias
- Quasi-randomized trial design
- Published abstracts without available full-text manuscript
- Studies assessing AV access that was never functional prior to intervention

#### Qualitative synthesis

All reported outcomes will be synthesized and reported in a qualitative manner. Furthermore, clinical outcomes will only be synthesized in a qualitative manner, as the expected heterogeneity in clinical situations and reporting will preclude quantitative analysis.

#### Meta-bias

In addition to individual study assessment of risk of bias, all studies will be evaluated for indications of meta-bias. We will search for preceding published or registered protocols prior to study publication and evaluate for selective outcome reporting. Potential for reporting bias will be assessed by a funnel plot of the primary outcome.

### Confidence in cumulative estimate

The cumulative estimates will be reported with estimated 95% confidence intervals from both fixed and random effects models. The quality of all outcomes will be judged subjectively as a consensus among study authors, using the standardized Grading of Recommendations Assessment, Development and Evaluation methodology.

## Discussion

The prevalence of hemodialysis and resulting treatment of failing AV access continues to become more common. The large clinical and economic implications of improving treatment for failing AV access have prompted extensive development of alternate procedures and technologies to maximize outcomes. However, each new device continues to be compared with POBA, likely in an effort to maximize the demonstrated relative effectiveness of the new technology without requiring large sample sizes. This has left clinicians with an array of potential tools to address failing AV access, but without evidence to judge the relative effectiveness between these multiple technologies. A network meta-analysis may allow us to use the existing evidence to compare treatments for failing AV access that have never been directly compared before.

The inherent limitations in managing bias and heterogeneity in any meta-analysis may be amplified in a network meta-analysis. Our study will minimize these sources of error through diligent assessment of risk of bias and qualitative and quantitative assessments of homogeneity. Nonetheless, occult underlying bias will persist as a limitation of this study. Should any amendments to the protocol be required, we will first require consensus by all listed authors. The amendment will be updated in the PROSPERO registration, and the nature and timing of the change will be explicitly described in the methods section of the final manuscript.

There appears to be a sufficient existing body of evidence to support a robust network meta-analysis on this topic. The results of this study will be of significant relevance to clinicians, researchers, and device companies who all participate in the ongoing efforts to limit post-intervention AV access failure.

## Data Availability

The datasets generated and analyzed during the current study will be available from the corresponding author on reasonable request.

## Appendix 1

### Proposed search syntax for MEDLINE, using OVID interface and including Cochrane Highly Sensitive RCT filter

Arteriovenous Access (OR)

- Arteriovenous Shunt, Surgical/
- (arterioven* adj3 fistula).tw.
- (arterioven* adj3 graft).tw.
- (AV adj3 fistula).tw.
- (AV adj3 graft).tw.
- (access* adj3 fistula).tw.
- (access* adj3 graft).tw.
- (dialys* adj3 fistula).tw.
- (dialys* adj3 graft).tw.
- (hemodialys* adj3 fistula).tw.
- (hemodialys* adj3 graft).tw.
- (dialysis adj3 access).tw.
- (hemodialysis adj3 access).tw.

Secondary Procedure (OR)

- Endovascular Procedures/
- Blood Vessel Prosthesis Implantation/
- surgery.tw.
- surgical.tw.
- Angioplasty/
- angioplast*.tw.
- Stents/
- stent*.tw.
- (cutting adj3 balloon).tw.
- (drug adj3 eluting).tw.
- Paclitaxel/
- paclitaxel.tw.
- Sirolimus/
- sirolimus.tw.
- Everolimus/
- everolimus.tw.

RCT filter (Cochrane Highly Sensitive Search Strategy for Identifying Randomized Trials: Sensitivity-and Precision-Maximizing Version [24]

1. randomized controlled trial.pt.
2. controlled clinical trial.pt.
3. randomized.ab.
4. placebo.ab.
5. clinical trials as topic.sh.
6. randomly.ab.
7. trial.ti.
8. 1 or 2 or 3 or 4 or 5 or 6 or 7
9. Exp animals/ not humans.sh.
10. 8 not 9

Final search: (Arteriovenous Access) AND (Secondary Procedure) AND (RCT Filter)

Limits:

- 1977 to present

## Appendix 2

### Proposed search syntax for EMBASE, using OVID interface and including Cochrane Handbook RCT filter

Arteriovenous Access (OR)

- (arterioven* adj3 fistula).tw.
- (arterioven* adj3 graft).tw.
- (AV adj3 fistula).tw.
- (AV adj3 graft).tw.
- (access* adj3 fistula).tw.
- (access* adj3 graft).tw.
- (dialys* adj3 fistula).tw.
- (dialys* adj3 graft).tw.
- (hemodialys* adj3 fistula).tw.
- (hemodialys* adj3 graft).tw.
- (dialysis adj3 access).tw.
- (hemodialysis adj3 access).tw.

Secondary Procedure (OR)

- Surgery/
- surgery.tw.
- surgical.tw.
- Angioplasty/
- angioplast*.tw.
- Stent/
- stent*.tw.
- (cutting adj3 balloon).tw.
- (drug adj3 eluting).tw.
- Paclitaxel/
- paclitaxel.tw.
- Rapamycin/
- sirolimus.tw.
- Everolimus/
- everolimus.tw.

Cochrane Handbook EMBASE RCT Filter (OR)

11. ‘crossover procedure’.de.
12. ‘double-blind procedure’.de.
13. ‘randomized controlled trial’.de.
14. ‘single-blind procedure’.de.
15. Random*.tw.
16. Factorial*.tw.
17. Crossover*.tw.
18. (Cross adj2 over*).tw.
19. Placebo*.tw.
20. (doubl* adj2 blind*).tw.
21. (singl* adj2 blind*).tw.
22. Assign*.tw.
23. Allocat*.tw.
24. Voluneer*.tw.

Final search: (Arteriovenous Access) AND (Secondary Procedure) AND (RCT Filter)

Limits:

- 1977 to present

## Abbreviations

AV: Arteriovenous
HD: Hemodialysis
KDIGO: Kidney Disease: Improving Global Outcomes
AVF: Arteriovenous fistula
ITT: Intention to treat
POBA: Plain old balloon angioplasty
